# Development and optimization of processing techniques for intermediate moisture muskmelon chunks

**DOI:** 10.1002/fsn3.1183

**Published:** 2019-08-30

**Authors:** Ahmad Din, Muhammad Nadeem, Farhan Saeed, Muhammad Haseeb Ahmad, Tabussam Tufail, Huma Bader Ul Ain, Zarina Mushtaq, Shahzad Hussain, Faqir Muhammad Anjum

**Affiliations:** ^1^ Ayub Agricultural Research Institute Faisalabad Pakistan; ^2^ Institute of Food Science & Nutrition University of Sargodha Sargodha Pakistan; ^3^ Institute of Home & Food Sciences Government College University Faisalabad Pakistan; ^4^ College of Food and Agricultural Sciences King Saud University Riyadh Saudi Arabia; ^5^ The University of the Gambia Serekunda Gambia

**Keywords:** intermediate moisture, muskmelon, physiochemical tests, sensory evaluation, storage behavior

## Abstract

Muskmelon (*Cucumis melo* ***)*** fruit is a best source of vitamins, minerals, and bioactive components. Ingestion of high sugary drinks leads to numerous ailments such as diabetes mellitus, obesity, and tooth decay. This study intended at formulation of intermediate moisture food with various combinations of sugar and glycerol and same levels of potassium metabisulphite, potassium sorbate, calcium chloride, and citric acid. It was observed a gradual decrease in TSS (56.53–53.28), vitamin C level in all treatments with the passage of time. The declining trend in ascorbic acid (25.49–21.63 mg/100 g) content of muskmelon chunks was increased as a function of storage. Sensory results showed that there was declining trend in the scores obtained for color parameter, that is, *L* ^*^ from 60.23 to 55.98. The overall results showed that combination of different additives contributed best values (T_3_) for flavor (7.70), taste (8.15), vitamin C (25.60 mg/100 g), and pH (5.16) as compared to other treatments. Conclusively, developed chunks of treatment 3 are physicochemical and organoleptically considered best, as it is helpful to sustain life stability of muskmelon and enhance its marketability.

## 1 INTRODUCTION

Muskmelon (*Cucumis melo*) is most consumable fruit throughout the world due to nice taste, flavor, texture, and beneficial effect on human health. It has 4th position in fresh fruit market and good source of nutrients having various varieties (Raji et al., 2018; Mabalaha, Mitei, & Yeboah, 2007). The cucurbitaceae family contains important cultivars, that is, cantaloupe, casaba, honey dew with netted, plain surfaces, and yellow to greenish external skin colors having impressive amount of vitamin C and vitamin A contents (Phisut, Rattanawedee, & Aekkasak, 2013). The functional components present in cantaloupe are vitamins, minerals, and pigments, which are responsible for health benefits like antioxidants and anti‐inflammatory properties. The consumption of muskmelon in United States was 1.18 billion kg after apples, bananas, and watermelons during the crop year 2010 (USDA‐ERS, 2010). The previous studies suggested that intermediate‐infrared radiation would be an effective method in industrial drying of fruit pomace alternative to hot air drying Zhou et al. (2019). The preferences in terms of acceptance by the consumers for melons are due to texture, flavor, and sweetness intensity (Lester, 2006). The two major cultivated and approved muskmelon varieties are Ravi and T‐96, which are famous for nice texture, aroma with good sweetness level varying from 8.00 to 14.00 °brix in Pakistan.

It has been reported that about 30%–50% of fruits and vegetables are wasted after harvesting during transportation, storage, and processing in developing countries (Alzamora, Tapia, & Lopez, 2000). During 2015–16, in Pakistan, the annual production of fruits and vegetables was 17.35 million tonnes (GOP, 2015–16). The enzymes present in fruits and vegetables are polyphenol oxidase which is responsible for browning of cut surfaces to catalyze the oxidation of phenolic compounds (Whitaker & Lee, 1995). After harvesting, the life of cantaloupe is not more than 2 weeks and this is great loss in the form of crop and economic value (Solval, Sundararajan, Alfaro, & Sathivel, 2012). Drying is an old method of food preservation to increase the postharvest life through decreasing the bulk weight and water activity. Changes in color degradation, microbial growth, changes in texture, and loss in nutrients are commonly observed during storage of dried fruit products (Salunke, Bolin, & Reddy, 1991). In case of osmotic dehydration, the excess moisture is partially expelled from the food by placing it in hypertonic sugar sirup. The intermediate moisture level products are good in nutritional values, easily consumable, reduce cost of transportation, and storage (Asavasanti, Tantipaibulvut, Samaal, & Sanuksaen, 2018; Corzo & Gomes, 2004; Leistner, 1992). It is an important food preservation process to decrease the input cost of energy consumption, enhance the food quality and as a pretreatment slow down enzymatic reaction, preserve natural color, and volatile aromas (Pokharkar, Prasad, & Das, 1997; Torreggiani, 1993). The main purpose of this research project was to develop and optimize the processing techniques for intermediate level muskmelon chunks and to assess the shelf stability through quality and sensory evaluation during storage period.

## 2 MATERIALS AND METHODS

### 2.1 Chemicals reagents and glassware

The chemicals, reagents, and glassware used for analytical purpose were purchased from authorized dealers of Merck, Sigma‐Aldrich, Riedel de Haen, and Pyrex from local scientific store market of Faisalabad, Pakistan.

### 2.2 Muskmelon pretreatment and samples preparation

Muskmelon (*C. melo*) varieties, that is, Ravi and T‐96, were procured from the research area of Vegetable Research Institute, Ayub Agricultural Research Institute Faisalabad, Pakistan. The samples for R&D studies were manually sorted from the diseased, injured, and bruised one. The selected healthy, uniform in shape samples were washed with fresh tap water and then manually removed the seeds, peeled and cut into small pieces with dimension, that is, 3.0 × 2.0 × 0.5 cm chunks with sharp knife. Before osmo dehydration; the muskmelon samples were dipped in 2.0% CaCl_2_ for 15 min and drained.

### 2.3 Procedure for osmo dehydration and storage

The sugar solutions of 30 °brix were prepared by adding the preservatives with and without glycerol according to the treatment plan (Table1). The pretreated samples were immersed in sugars solutions for 24 hr. After immersion in sugar solution for 24 hr, samples were removed and drained. Then, the samples were placed in dehydrator (tray dryer Model No. R‐5A, commercial dehydrator systems, USA) at 60°C up to water activity (a_w_) 0.60 monitored with water activity meter (Rotronic Hygropalm Model: A_w_‐DIO, Rotronic Instrument Corp) throughout the drying process. After retaining the required level of moisture level through water activity meter, 50 g muskmelon chunks were packed in high density polyethylene bags, sealed, and stored at ambient storage condition of laboratory (25 ± 2°C). The prepared samples were given name as intermediate level muskmelon chunks in this research paper, which were evaluated after 1 month up to 3‐month storage period with various analyses for quality and sensory attributes.

**Table 1:** Treatment plan

Treatments	Muskmelon varieties	CaCl_2_ (%) treatment before drying	Sugar (%)	Glycerol (%)	KMS (%)	K. sorbate (%)	Citric acid (%)
T_1_	Ravi	2.0	30.0	0.0	0.20	0.40	0.20
T_2_	Ravi	2.0	15.0	15.0	0.20	0.40	0.20
T_3_	T‐96	2.0	30.0	0.0	0.20	0.40	0.20
T_4_	T‐96	2.0	15.0	15.0	0.20	0.40	0.20

## 3 PHYSICOCHEMICAL TEST

The raw materials and the prepared intermediate moisture muskmelon samples were analyzed in triplicate for various quality and sensory attribute tests according to their respective methods as given below:

### 3.1 TSS

Total soluble solids were determined by digital refractometer (Model: HI 96801, HANNA instruments) with a measurement range from 0 to 85 °brix. The results for intermediate moisture muskmelon chunks were described in TSS (°brix) according to AOAC (1990).

### 3.2 pH

The pH of samples was determined by following method as given by Ranganna (1999) in which 20g sample was ground in distilled water (100 ml) and filtered it. The pH meter (Model: HI 2211, HANNA instruments) was calibrated with buffer solutions at 4, 7, and 10 at room temperature and noted the reading of filtrate from display.

### 3.3 Total acidity

The acidity of samples was measured according to the method as explained by Ranganna (1999). Two gram grounded sample was taken in 10 ml distilled water and filtered it. Add two to three drops of phenolphthalein as an indicator in filtrate and titrated against 0.1N NaOH till light pink color end point, noted the titer value and calculated the total acidity. The obtained results were expressed in percentage.

### 3.4 Vitamin C

The oxidized amount of vitamin C was calculated by adopting the titrimetric method Ranganna (1999) in which 2,6‐dichlorophenolindophenol reagent reduction values were recorded. The results were expressed as mg/100 g loss in vitamin C during storage period.

### 3.5 Color measurement

The color of intermediate moisture muskmelon chunks was determined at different storage intervals according to method explained by Rocha and Morais (2003) with a handheld tristimulus reflectance color meter (PCM/PSM model, Color‐Tec). The color values were recorded using a CIE–L^*^a^*^b^*^ uniform color space (−*Lab*), where color parameters showed lightness for L^*^ while a^*^ indicates redness on a green (−) to red (+) axis, and b^*^ parameter for yellowness on a blue (−) to yellow (+) axis.

### 3.6 Water activity

The water activity was measured by filling the sample cup of water activity meter (Rotronic Hygropalm Model: A_w_‐DIO, Rotronic Instrument Corp) at room temperature. The results were expressed as water activity (a_w_) of intermediate level muskmelon chunks.

### 3.7 Textural analysis

The texture of muskmelon samples was measured by adopting the method as given by Piga et al. (2005) with minor modifications. Puncture test was performed to measure the texture values with texture analyzer (Model: TA‐XT2, Stable Microsystems) having capacity of 5 kg load cell equipped with texture expert program version 4.0.9.0 for analyzing the data. Intermediate level muskmelon samples were punctured to measure the firmness from external to internal surface tissue cells by placing the samples in the center beneath the needle probe. Before the performance of test, load cell and probe were calibrated and measured the puncturing force (g) against time (s).

### 3.8 Sensory evaluation

The samples of intermediate moisture muskmelon chunks were organoleptically evaluated for different parameters like color, taste, texture, chewability, and overall acceptability. A panel of five trained judges from the Food Technology Section, Ayub Agricultural Research Institute, Faisalabad, were selected and presented the samples randomly after each storage interval according to the method as given by Harry and Heymann (2010). The score varies from extremely liked nine points to extremely dislike one point according to the provided scoring proforma Appendix S1. The significance level was presented as *p* < .05.

### 3.9 Statistical analysis

The collected data in replicated form were subjected to statistical analysis by using the software statistix 8.1 and compare the results through one‐way ANOVA and CRD factorial to determine the level of significance, and the results were elaborated in means and standard deviations (*SD*) as mentioned by Saxena, Mishra, Chander, and Sharma (2009). The means with significant difference were presented as *p* < .05 and indicated with different letters.

## 4 RESULTS AND DISCUSSION

### 4.1 TSS

**Figure 1:**
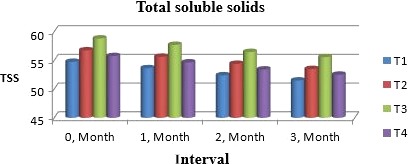
Mean values for total soluble solids of Muskmelon chunks: T1 = Ravi (Variety), 2% (CaCl_2_), 30% (Sugar) 0% (Glycerol), 0.20% (KMS), 0.40% (K. sorbate), 0.20% (Citric Acid). T2 = Ravi (Variety), 2% (CaCl_2_), 15% (Sugar) 15% (Glycerol), 0.20% (KMS), 0.40% (K. sorbate), 0.20% (Citric Acid). T3 = T‐96 (Variety), 2% (CaCl_2_), 30% (Sugar) 0% (Glycerol), 0.20% (KMS), 0.40% (K. sorbate), 0.20% (Citric Acid). T4 = T‐96 (Variety), 2% (CaCl_2_), 15% (Sugar) 15% (Glycerol), 0.20% (KMS), 0.40% (K. sorbate), 0.20% (Citric Acid)

Total soluble solids undergo changes in muskmelon chunks due to moisture because they picked moisture and decreased significantly the TSS. Figure 1 indicates that at first month of storage, it ranged from 53.08d to 54.09c from T_1_ to T_4_, with *p* ≤ .05. According to Beaulieu and Lea (2010), likewise pattern was observed during storage of muskmelon when dried as intermediate moisture food. These results are in accordance with Chen, Chan, and Li (2011) who analyzed melon powder for storage stability and checked its feasibility for further use.

### 4.2 T.A. (%)

Titratable acidity plays a crucial role in the shelf life of any type of fruit or drink and inhibits growth of microorganisms in foods. Titratable acidity (%) changed slightly during storage (Figure 1). Values ranged from 0.223b to 0.233a from T_2_ to T_4_. Likewise, Souza et al. (2009) explained that melon chunks can be used as additive in food formulations as its titratable acidity remains same (0.33 before and 0.34 after). The similar trend of acidity (0.26–0.39) was observed by Majumdar, Vasudish, Premavalli, and Bawa (2008) during storage period of mint leaf juice. Increase in acidity is attributed to sugar degradation as a result of enzymatic action.

### 4.3 pH

**Figure 2:**
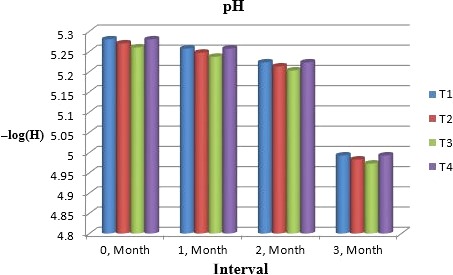
Mean values for pH of Muskmelon chunks: T1 = Ravi (Variety), 2% (CaCl_2_), 30% (Sugar) 0% (Glycerol), 0.20% (KMS), 0.40% (K. sorbate), 0.20% (Citric Acid). T2 = Ravi (Variety), 2% (CaCl_2_), 15% (Sugar) 15% (Glycerol), 0.20% (KMS), 0.40% (K. sorbate), 0.20% (Citric Acid). T3 = T‐96 (Variety), 2% (CaCl_2_), 30% (Sugar) 0% (Glycerol), 0.20% (KMS), 0.40% (K. sorbate), 0.20% (Citric Acid). T4 = T‐96 (Variety), 2% (CaCl_2_), 15% (Sugar) 15% (Glycerol), 0.20% (KMS), 0.40% (K. sorbate), 0.20% (Citric Acid)

pH has inverse relation with acidity in any medium as shown in Figure 2. The present study for muskmelon chunk also expressed the same pattern of pH. pH values depicted the range 5.188a to 5.188a from T_1_ to T_4_. With the passage of time, pH showed a decreasing tendency and was 5.273a, 5.249b, 5.216c, and 4.986d for T_1_, T_2_, T_3_, and T_4_, respectively. This decrease may be due to in situ acid production in product. According to Beaulieu and Lea (2010), similar trend was elucidated during storage of muskmelon drink. Dhaliwal and Hira (2004) studied carrot–spinach and pineapple juices and expressed that 3‐month storage cause decrease in pH. Likewise, Hussain, Zaib, and Ayub (2010) also observed that storage of apricot and apple blend showed slight decrease in pH.

### 4.4 Ascorbic acid (mg/100 ml)

The vitamin C content is reduced significantly during storage interval. Ascorbic acid amount decreased from 23.933 to 20.073 for T_1_, 26.023 to 22.160 for T_2_, 27.190 to 23.330 for T_3_, and 24.813 to 21.629d for T_4_ with the passage of time. These current results are in correspondence with the research work of Pruthi et al. (2010) who prepared kinnow‐malta juice. Similar trend in ascorbic acid degradation was also observed by Bhardwaj and Mukherjee (2012) who prepared orange juice and studies effect of storage on physicochemical parameters.

### 4.5 Texture

**Figure 3:**
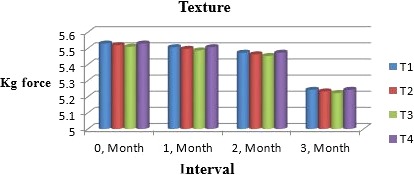
Mean values for texture of Muskmelon chunks: T1 = Ravi (Variety), 2% (CaCl_2_), 30% (Sugar) 0% (Glycerol), 0.20% (KMS), 0.40% (K. sorbate), 0.20% (Citric Acid). T2 = Ravi (Variety), 2% (CaCl_2_), 15% (Sugar) 15% (Glycerol), 0.20% (KMS), 0.40% (K. sorbate), 0.20% (Citric Acid). T3 = T‐96 (Variety), 2% (CaCl_2_), 30% (Sugar) 0% (Glycerol), 0.20% (KMS), 0.40% (K. sorbate), 0.20% (Citric Acid). T4 = T‐96 (Variety), 2% (CaCl_2_), 15% (Sugar) 15% (Glycerol), 0.20% (KMS), 0.40% (K. sorbate), 0.20% (Citric Acid)

Muskmelon chunk hardness increases with the storage if packs are intact but when chunks pick moisture harness is reduced little bit. Calcium chloride increases hardness due to cross‐links of carboxyl carbon with polyuronide chains and calcium ion. Sarolia and Mukherjee (2008) proved that texture of mango leather was improved by adding calcium chloride and did not change during storage to greater extent. According to Nchez‐moreno, Plaza, Adeancos, and Pilarcano (2003) texture (viscosity) of orange juice can be maintained by adding calcium salts during subsequent storage. Moreover, Figure 3 shows that keeping quality of melon chunks greatly depends upon their texture during shelf periods. Softness in texture of melon was observed by Lamikanra and Watson (2009) who explained it due to loosening of cross‐links in structure of melon but texture was improved by adding calcium sorbate.

### 4.6 Water activity

**Figure 4:**
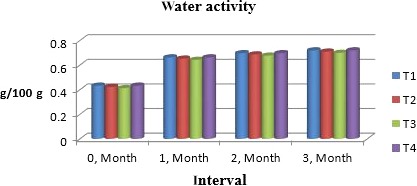
Mean values for water activity of Muskmelon chunks: T1 = Ravi (Variety), 2% (CaCl_2_), 30% (Sugar) 0% (Glycerol), 0.20% (KMS), 0.40% (K. sorbate), 0.20% (Citric Acid). T2 = Ravi (Variety), 2% (CaCl_2_), 15% (Sugar) 15% (Glycerol), 0.20% (KMS), 0.40% (K. sorbate), 0.20% (Citric Acid). T3 = T‐96 (Variety), 2% (CaCl_2_), 30% (Sugar) 0% (Glycerol), 0.20% (KMS), 0.40% (K. sorbate), 0.20% (Citric Acid). T4 = T‐96 (Variety), 2% (CaCl_2_), 15% (Sugar) 15% (Glycerol), 0.20% (KMS), 0.40% (K. sorbate), 0.20% (Citric Acid)

Shelf life of any product depends upon water activity, so it is important to maintain it at specific level. Figure 4 shows that water activity of chunks was increased during storage due to slight pick of moisture during storage. According to study of Dhingra, Singh, Patil, and Uppal (2008), osmotic drying of fruit and vegetables water activity is lowered and maintained in storage if additives like glucose and humectants are added. Likewise, pattern was also observed by Rangana (2012) who analyzed and stabilized number of fruits during storage. Produced the similar results and proved this by using apple chunks.

### 4.7 Sensory evaluation

**Figure 5:**
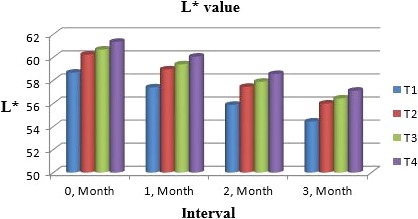
Mean values for color (*L* ^*^) of Muskmelon chunks: T1 = Ravi (Variety), 2% (CaCl_2_), 30% (Sugar) 0% (Glycerol), 0.20% (KMS), 0.40% (K. sorbate), 0.20% (Citric Acid). T2 = Ravi (Variety), 2% (CaCl_2_), 15% (Sugar) 15% (Glycerol), 0.20% (KMS), 0.40% (K. sorbate), 0.20% (Citric Acid). T3 = T‐96 (Variety), 2% (CaCl_2_), 30% (Sugar) 0% (Glycerol), 0.20% (KMS), 0.40% (K. sorbate), 0.20% (Citric Acid). T4 = T‐96 (Variety), 2% (CaCl_2_), 15% (Sugar) 15% (Glycerol), 0.20% (KMS), 0.40% (K. sorbate), 0.20% (Citric Acid)

**Figure 6:**
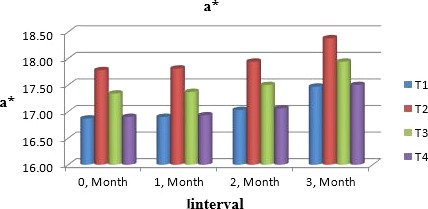
Mean values for color (*a* ^*^) of Muskmelon chunks: T1 = Ravi (Variety), 2% (CaCl_2_), 30% (Sugar) 0% (Glycerol), 0.20% (KMS), 0.40% (K. sorbate), 0.20% (Citric Acid). T2 = Ravi (Variety), 2% (CaCl_2_), 15% (Sugar) 15% (Glycerol), 0.20% (KMS), 0.40% (K. sorbate), 0.20% (Citric Acid). T3 = T‐96 (Variety), 2% (CaCl_2_), 30% (Sugar) 0% (Glycerol), 0.20% (KMS), 0.40% (K. sorbate), 0.20% (Citric Acid). T4 = T‐96 (Variety), 2% (CaCl_2_), 15% (Sugar) 15% (Glycerol), 0.20% (KMS), 0.40% (K. sorbate), 0.20% (Citric Acid)

**Figure 7:**
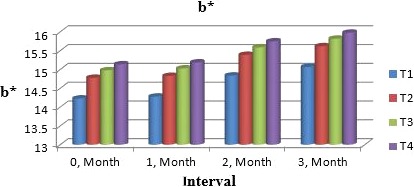
Mean values for color (*b* ^*^) of Muskmelon chunks: T1 = Ravi (Variety), 2% (CaCl_2_), 30% (Sugar) 0% (Glycerol), 0.20% (KMS), 0.40% (K. sorbate), 0.20% (Citric Acid). T2 = Ravi (Variety), 2% (CaCl_2_), 15% (Sugar) 15% (Glycerol), 0.20% (KMS), 0.40% (K. sorbate), 0.20% (Citric Acid). T3 = T‐96 (Variety), 2% (CaCl_2_), 30% (Sugar) 0% (Glycerol), 0.20% (KMS), 0.40% (K. sorbate), 0.20% (Citric Acid). T4 = T‐96 (Variety), 2% (CaCl_2_), 15% (Sugar) 15% (Glycerol), 0.20% (KMS), 0.40% (K. sorbate), 0.20% (Citric Acid)

**Figure 8:**
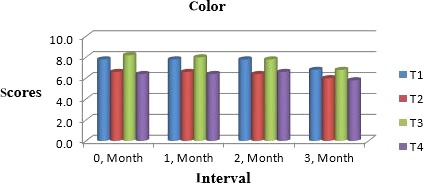
Mean values for color of Muskmelon chunks: T1 = Ravi (Variety), 2% (CaCl_2_), 30% (Sugar) 0% (Glycerol), 0.20% (KMS), 0.40% (K. sorbate), 0.20% (Citric Acid). T2 = Ravi (Variety), 2% (CaCl_2_), 15% (Sugar) 15% (Glycerol), 0.20% (KMS), 0.40% (K. sorbate), 0.20% (Citric Acid). T3 = T‐96 (Variety), 2% (CaCl_2_), 30% (Sugar) 0% (Glycerol), 0.20% (KMS), 0.40% (K. sorbate), 0.20% (Citric Acid). T4 = T‐96 (Variety), 2% (CaCl_2_), 15% (Sugar) 15% (Glycerol), 0.20% (KMS), 0.40% (K. sorbate), 0.20% (Citric Acid)

**Figure 9:**
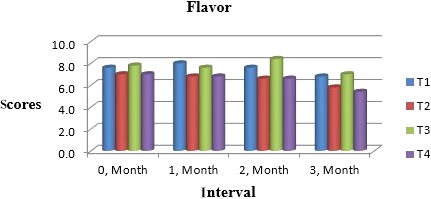
Mean values for flavor of Muskmelon chunks: T1 = Ravi (Variety), 2% (CaCl_2_), 30% (Sugar) 0% (Glycerol), 0.20% (KMS), 0.40% (K. sorbate), 0.20% (Citric Acid). T2 = Ravi (Variety), 2% (CaCl_2_), 15% (Sugar) 15% (Glycerol), 0.20% (KMS), 0.40% (K. sorbate), 0.20% (Citric Acid). T3 = T‐96 (Variety), 2% (CaCl_2_), 30% (Sugar) 0% (Glycerol), 0.20% (KMS), 0.40% (K. sorbate), 0.20% (Citric Acid). T4 = T‐96 (Variety), 2% (CaCl_2_), 15% (Sugar) 15% (Glycerol), 0.20% (KMS), 0.40% (K. sorbate), 0.20% (Citric Acid)

**Figure 10:**
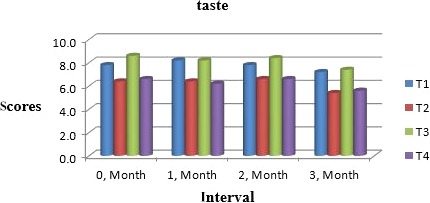
Mean values for taste of Muskmelon chunks: T1 = Ravi (Variety), 2% (CaCl_2_), 30% (Sugar) 0% (Glycerol), 0.20% (KMS), 0.40% (K. sorbate), 0.20% (Citric Acid). T2 = Ravi (Variety), 2% (CaCl_2_), 15% (Sugar) 15% (Glycerol), 0.20% (KMS), 0.40% (K. sorbate), 0.20% (Citric Acid). T3 = T‐96 (Variety), 2% (CaCl_2_), 30% (Sugar) 0% (Glycerol), 0.20% (KMS), 0.40% (K. sorbate), 0.20% (Citric Acid). T4 = T‐96 (Variety), 2% (CaCl_2_), 15% (Sugar) 15% (Glycerol), 0.20% (KMS), 0.40% (K. sorbate), 0.20% (Citric Acid)

**Figure 11:**
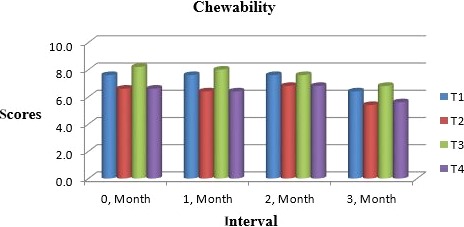
Mean values for chewability of Muskmelon chunks: T1 = Ravi (Variety), 2% (CaCl_2_), 30% (Sugar) 0% (Glycerol), 0.20% (KMS), 0.40% (K. sorbate), 0.20% (Citric Acid). T2 = Ravi (Variety), 2% (CaCl_2_), 15% (Sugar) 15% (Glycerol), 0.20% (KMS), 0.40% (K. sorbate), 0.20% (Citric Acid). T3 = T‐96 (Variety), 2% (CaCl_2_), 30% (Sugar) 0% (Glycerol), 0.20% (KMS), 0.40% (K. sorbate), 0.20% (Citric Acid). T4 = T‐96 (Variety), 2% (CaCl_2_), 15% (Sugar) 15% (Glycerol), 0.20% (KMS), 0.40% (K. sorbate), 0.20% (Citric Acid)

**Figure 12:**
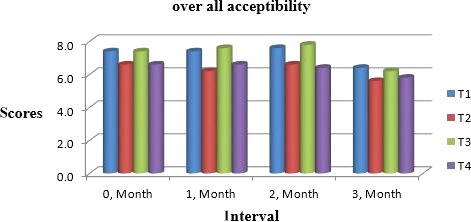
Mean values for overall acceptability of Muskmelon chunks: T1 = Ravi (Variety), 2% (CaCl_2_), 30% (Sugar) 0% (Glycerol), 0.20% (KMS), 0.40% (K. sorbate), 0.20% (Citric Acid). T2 = Ravi (Variety), 2% (CaCl_2_), 15% (Sugar) 15% (Glycerol), 0.20% (KMS), 0.40% (K. sorbate), 0.20% (Citric Acid). T3 = T‐96 (Variety), 2% (CaCl_2_), 30% (Sugar) 0% (Glycerol), 0.20% (KMS), 0.40% (K. sorbate), 0.20% (Citric Acid). T4 = T‐96 (Variety), 2% (CaCl_2_), 15% (Sugar) 15% (Glycerol), 0.20% (KMS), 0.40% (K. sorbate), 0.20% (Citric Acid)

Muskmelon chunks were evaluated organoleptically during 3 months of storage. The evaluation was conducted after 1‐month interval by a panel of professional judges on the basis of nine‐point hedonic scale. The statistical results revealed that all sensory characteristics differ significantly (*p* ≤ .05) with regard to treatments as well as during storage as shown in Figures 10 and 11. The treatment T_3_ was the highly ranked for all aspects of organoleptic evaluation. The T_2_ treatment was the next best ranked treatment. The least ranked treatment by the panelists was control followed by T_4_. The scores for flavor and taste were declining through storage intervals due to enclosure of compounds which are used for shelf stability. Color changes during storage also presented decreasing trend in all samples that may be due to storage conditions depicted in Figures 5, 6, 7, 8. A steady reduction in flavor may be due to degradation of flavor during storage as shown in Figure 9. Taste and chewability is shown in Figures 10 and 11. Such reasons for decrease in flavor have been reported by who studied fresh cuts of muskmelon. Figure 12 scores for overall acceptability which were cumulative effect of all other sensory parameters presented that during storage period of 3 months, there was small reduction and among all the treatments best scores were observed by T_3_. Similar tendency in sensorial properties of ice tea was observed by Bhardwaj and Mukherjee (2012) as a function of storage.

## 5 CONCLUSION

A novel intermediate moisture product having better storage stability was developed. Developed chunks of treatment 3 are considered best physicochemically and organoleptically. It has been established from the current investigation that to sustain life stability of muskmelon and enhance its marketability, we can dry them and add sugar, potassium sorbate, calcium chloride, and potassium metabisulphite at specific level. Future explorations would be required in this respect to discover more unique methods and processing conditions. Unique methods called osmodehyration and vacuum coupled dehydration can be used for formation of intermediate foods. Moreover, we can extract bioactive components from intermediate moisture foods as this can be accommodated easily in supercritical fluid extractor.

## CONFLICT OF INTEREST

Authors declare that they have no conflict of interests.

## STATEMENT OF DECLARATION FOR HUMAN SUBJECTS

This study has no involvement of human or animal subjects.

## References

[fsn31183-bib-0001] Alzamora, S. E. , Tapia, M. S. , & Lopez, M. (2000). Minimally processed fruit and vegetables. Fundamental aspects and applications (pp. 21–181). Frederick, MD: Aspen Publishing Company Inc.

[fsn31183-bib-0002] AOAC (Association of Official Analytical Chemistry) (1990). Official methods of analysis 2 (13th ed., pp. 46–987). Rockville, MD: AOAC.

[fsn31183-bib-0003] Asavasanti, S. , Tantipaibulvut, S. , Samaal, A. , & Sanuksaen, W. (2018). Efficiency improvement of bioactive compounds extraction from cantaloupe and muskmelon by freeze‐thawing and PEF. Research Journal Phranakhon Rajabhat: Science and Technology, 13(1), 50–63.

[fsn31183-bib-0004] Beaulieu, J. C. , & Lea, J. M. (2010). Aroma volatile differences in commercial orange fleshed cantaloupes, the inbred parental lines, and stored fresh‐cuts. Acta Horticulturae, 628, 809–815.

[fsn31183-bib-0005] Bhardwaj, R. L. , & Mukherjee, S. (2012). Factors affecting physico‐chemical, sensory and microbiological quality of Kinnow juice blends. Journal of Nutrition and Food Science, 2, 6 10.4172/2155-9600.1000148

[fsn31183-bib-0006] Chen, Q. , Chan, L. L. Y. , & Li, E. T. S. (2011). Bitter melon (*Momordica charantia*) reduces adiposity, lowers serum insulin and normalizes glucose tolerance in rats fed a high fat diet. Journal of Nutrition, 2011(133), 1088–1093. 10.1093/jn/133.4.1088 12672924

[fsn31183-bib-0007] Corzo, O. , & Gomes, E. R. (2004). Optimization of osmotic dehydration of cantaloupe using desired function methodology. Journal of Food Engineering, 64, 213–219. 10.1016/j.jfoodeng.2003.09.035

[fsn31183-bib-0008] Dhaliwal, M. , & Hira, C. K. (2004). Effect of storage on physico‐chemical and nutritional characteristics of carrot‐spinach and carrot‐pineapple juices. Journal of Food Science and Technology, 41, 613–617.

[fsn31183-bib-0009] Dhingra, D. , Singh, J. , Patil, P. , & Uppal, D. S. (2008). Osmotic dehydration of fruits and vegetables: A review. Journal of Food Science and Technology. 45: 209-217. 10.1007/s13197-012-0659-2 PMC4152536 25190823

[fsn31183-bib-0011] Harry, T. L. , & Heymann, H. (2010). Sensory evaluation of food: Principles and practices (2nd ed.). New York, NY/Dordrecht, The Netherlands/Heidelberg, Germany/London, UK: Springer.

[fsn31183-bib-0012] Hussain, I. , Zaib, A. , & Ayub, M. (2010). Quality attributes of apple and apricot blend juice preserved with potassium sorbate during storage at low temperature. International Journal of Food Science, 12, 80–86.

[fsn31183-bib-0013] Lamikanra, O. , & Watson, M. A. (2009). Temperature and storage duration effects on esterase activity in fresh‐cut cantaloupe melon. Journal of Food Science, 68, 790–793. 10.1111/j.1365-2621.2003.tb08243.x

[fsn31183-bib-0014] Leistner, L. (1992). Food preservation by combined methods. Food Research International, 25, 151–158. 10.1016/0963-9969(92)90158-2

[fsn31183-bib-0015] Lester, G. (2006). Consumer preference quality attributes of melon fruits. Acta Horticulture, 712, 175–182. 10.17660/ActaHortic.2006.712.17

[fsn31183-bib-0016] Mabaleha, M. B. , Mitei, Y. C. , & Yeboah, S. O. (2007). A comparative study of the properties of selected melon seed oils as potential candidates for development into commercial edible vegetable oils. Journal of the American oil chemists' society, 84(1), 31–36.

[fsn31183-bib-0017] Majumdar, T. K. , Vasudish, C. R. , Premavalli, K. S. , & Bawa, A. S. (2008). Studies on processing and storage stability of ashgourd‐mint leave juice. Journal of Food Processing and Preservation, 34, 549–556.

[fsn31183-bib-0018] Nchez‐moreno, C. , Plaza, L. , Adeancos, B. , & Pilarcano, M. (2003). Vitamin C, Provitamin A, carotenoids and other carotenoids in high‐pressurized orange juice during refrigerated storage. Journal of Agricultural and Food Chemistry, 51, 647–653. 10.1021/jf020795o 12537436

[fsn31183-bib-0020] Phisut, N. , Rattanawedee, M. , & Aekkasak, K. (2013). Effect of osmotic dehydration process on the physical, chemical and sensory properties of osmo‐dried cantaloupe. International Food Research Journal, 20(1), 189–196.

[fsn31183-bib-0021] Piga, A. , Catzeddu, P. , Farris, S. , Roggio, T. , Sanguinetti, A. , & Scano, E. (2005). Textural evaluation of “Amretti” cooking during storage. Food Research and Technology, 221, 387–391.

[fsn31183-bib-0022] Pokharkar, S. M. , Prasad, S. , & Das, H. (1997). A model of osmotic concentration of banana slices. Journal of Food Science and Technology, 34, 230–232.

[fsn31183-bib-0023] Pruthi, J. S. , Manna, J. K. , Tectia, M. S. , Radhakriahna, S. G. , Eipeson, W. E. , Saroja, S. , & Chikkappaji, A. (2010). Studies on the utilization of Kinnow and Malta orange. Journal of Food Science and Technology, 21(3), 121–127.

[fsn31183-bib-0024] Raji, M. R. , Lotfi, M. , Tohidfar, M. , Zahedi, B. , Carra, A. , Abbate, L. , & Carimi, F. (2018). Somatic embryogenesis of muskmelon (*Cucumis melo* L.) and genetic stability assessment of regenerants using flow cytometry and ISSR markers. Protoplasma, 255(3), 873–883. 29248969 10.1007/s00709-017-1194-9

[fsn31183-bib-0025] Rangana, S. (2012). Handbook of analysis and quality control for fruits and vegetables products. New Delhi, India: Tata Mc‐Graw Hill Publishing Company Limited.

[fsn31183-bib-0026] Ranganna, S. (1999). Handbook of analysis and quality control for fruit and vegetable products. New Delhi: McGraw-Hill Education.

[fsn31183-bib-0027] Rocha, A. M. C. N. , & Morais, A. M. M. B. (2003). Shelf life of minimally processed apple (cv. Jonagored) determined by colour changes. Food control, 14(1), 13-20.

[fsn31183-bib-0028] Salunke, D. K. , Bolin, H. R. , & Reddy, N. R. . (1991). Storage, processing and nutritional quality of fruits and vegetables (Vol. ll, 2nd ed., pp. 21–105). Boca Raton, FL: CRC Press Inc.

[fsn31183-bib-0029] Sarolia, D. K. , & Mukherjee, S. (2008). Comparative efficiency of different preservation methods in keeping quality of lime (*Citrus aurantifolia*) swingle juice during storage. Haryana Journal of Horticultural Sciences, 31(3–4), 185–188.

[fsn31183-bib-0030] Saxena, S. , Mishra, B. B. , Chander, R. , & Sharma, A. (2009). Shelf stable intermediate moisture pineapple (*Ananas comosus*) slices using hurdle technology. LWT – Food Science and Technology, 42, 1681–1687. 10.1016/j.lwt.2009.05.009

[fsn31183-bib-0031] Solval, M. K. , Sundararajan, S. , Alfaro, L. , & Sathivel, S. (2012). Development of cantaloupe juice powder using spray drying technique. LWT – Food Science and Technology, 46, 287–293.

[fsn31183-bib-0032] Souza, A. S. , Borges, S. V. , Magalhães, N. F. , Ricardo, H. V. , Cereda, M. P. , & Daiuto, E. R. (2009). Influence of spray drying conditions on the physicochemical properties of dried pulptomato. Ciência E Tecnologia De Alimentos, 29, 291–294.

[fsn31183-bib-0033] Torreggiani, D. (1993). Osmotic dehydration in fruit and vegetable processing. Food Research International, 26, 59–68. 10.1016/0963-9969(93)90106-S

[fsn31183-bib-0034] USDA‐ERS (2010). Vegetables and melons yearbook. Retrieved from http://usda.mannlib.cornell.edu/MannUsda/viewDocumentInfo.do?documentD=1212

[fsn31183-bib-0035] Whitaker, J. R. , & Lee, C. Y. (1995). Recent advances in chemistry of enzymatic browning In Lee C. Y., & Whitaker J. R. (Eds.), Enzymatic browning and its prevention (pp. 2–7). Washington, DC: ACS Publications. ACS Symposium Series 600.

[fsn31183-bib-0036] Zhou, M. , Li, C. , Bi, J. , Jin, X. , Lyu, J. , & Li, X. (2019). Towards understanding the enhancement of moisture diffusion during intermediate‐infrared drying of peach pomace based on the glass transition theory. Innovative Food Science & Emerging Technologies, 54, 143–151. 10.1016/j.ifset.2019.04.003

