# Supplementary material for: Development and optimization of processing techniques for intermediate moisture muskmelon chunks
Source: Food Sci Nutr. 2019 Aug 30;7(10):3253–60. doi: 10.1002/fsn3.1183 (PMC6804771; doi:10.1002/fsn3.1183)
Supplement: Supplementary file 1 [file FSN3-7-3253-s001.docx]

**APPENDIX I**

**HEDONIC SCALE (9 POINT)**

**PRODUCT: INTERMEDIATE MOISTURE MUSKMELON CHUNKS**

***DATE*: __________**

**NAME OF JUDGE: __________________________**

| **SAMPLE NAME** | **COLOR** | **TASTE** | **TEXTURE** | **CHEW ABILITY** | **OVERALL ACCEPTABILITY** |
| --- | --- | --- | --- | --- | --- |
| **T_1_** |  |  |  |  |  |
| **T_2_** |  |  |  |  |  |
| **T_3_** |  |  |  |  |  |
| **T_4_** |  |  |  |  |  |

**SIGNATURE**

**REMARKS (IF ANY) _________________________________________**

**_________________________________________**

**_________________________________________**

**KEY FOR RANKING:**

**Dislike extremely 1**

**Dislike very much 2**

**Dislike moderately 3**

**Dislike slightly 4**

**Neither dislikes nor like 5**

**Like slightly 6**

**Like moderately 7**

**Like very much 8**

**Like extremely 9**
